# Local government institutions in Ghana: Core partners in health and safety performance in the construction industry

**DOI:** 10.1016/j.heliyon.2023.e19423

**Published:** 2023-08-24

**Authors:** Benjamin Boahene Akomah, Prasanna Venkatesan Ramani

**Affiliations:** School of Civil Engineering, VIT University, Vellore, 632014, Tamil Nadu, India

**Keywords:** District assemblies, Ghanaian construction industry, Safety performance, Relative importance index, Multivariate analysis

## Abstract

The potential contribution of district assemblies to improving health and safety in Ghana's construction industry has not been investigated. This paper attempts to identify the local government responsibilities that could influence health and safety performance in the construction industry and to develop a confirmatory factor model based on these responsibilities. The paper adopted a positivist philosophy and a deductive methodology. A literature search was conducted to identify the local government factors that could potentially impact health and safety performance in the Ghanaian construction industry. These factors were adapted and designed into a questionnaire, and the generated questionnaires were distributed to respondents for their feedback. It was identified using the relative importance index that “the development of sanctions for violating occupational health and safety statutory obligations,” “the institution of local government occupational health and safety approval and certification for new projects,” and “the creation of occupational health and safety departments and committees that are adequately resourced to assist the Department of Factories Inspectorate” were the three most important roles that district assemblies could play as partners to improve health and safety in the construction industry. Conversely, the multivariate analysis identified the evaluation of the safety policies and risk management plans of contractors and suppliers as the most important role local government institutions could play in improving health and safety in the construction industry. The result of the study suggests that local government efforts, when given the needed attention and support, could advance health and safety in the industry. The practical implication of the study is that it identifies the roles of district assemblies that could enhance health and safety in Ghana's construction industry. All district assemblies should consider implementing these health and safety initiatives in their districts and ensuring they are regularly enforced and evaluated.

## 1 Introduction

The construction industry significantly contributes to the world's economy [1]. However, it is among the most dangerous sectors because of the complex nature of operations [2]. Despite its tremendous contribution, the industry is bedevilled with many issues that impact project stakeholders' objectives, and one such issue is health and safety performance [3]. Occupational health and safety issues in the construction industry continue to make global headlines [4]. [5] posited that it has become a disturbing subject for all stakeholders. Interestingly, the health and safety of workers are still being undermined, leading to physical damage to employees and financial losses [6,7]. According to Ref. [8], the industry accounts for 16% and 22% of the American and UK construction industries, respectively. The International Labour Organisation (ILO) reported that 2.3 million workers die each year as a result of occupational accidents [7], and 317 million work-related accidents occur each year [5]. [9] postulated that poor health and safety management and performance increase overall health and safety costs. Contrastingly [1], averred that improving health and safety performance has economic benefits and enhances productivity. Health and safety improvement is inevitable because it enhances workflow and protects lives [10,11]. [12] highlighted that health and safety performance in developing countries is poor, and Ghana is no exception. According to Refs. [13,14], laxity in enforcing health and safety is the primary cause of poor health and safety performance.

Health and safety laws in Ghana are enshrined in the Factories, Offices and Shops Act 1970 and the Labour Act 2003. However, the comprehensive nature of construction activities makes the provisions in these laws inadequate [15]. Though both laws refer to employers' and employees’ responsibilities, the complexity of construction projects and the surge in the use of technology in construction activities make the laws inefficient in dealing with matters of health and safety (H&S). Besides the insufficiency of existing laws, enforcement is also a big challenge [13,15,16]. H&S enforcement in Ghana primarily rests on the shoulders of the Department of Factories Inspectorate (DFI). [17] stated that enforcement of the law, no matter how inadequate or insufficient it is, serves as a deterrent. [15,18] admitted that the enormity of the workload of the Department of Factories Inspectorate is overwhelming. Currently, the efficiency and visibility of the DFI in many regions are nothing to write home about. It raises the question of what the local government (LG) or district assemblies (DAs) can do to help improve H&S because they have the administrative power to manage their jurisdictions and enact their bylaws [19]. To give a facelift to H&S performance in the construction industry, district assemblies (DAs) need to be integrated into whatever existing systems are available. [20] disclosed that the effectiveness of health and safety compliance and performance enhancement requires comprehensive H&S policies that reflect the letter of existing laws and those yet to be enacted. In the United Kingdom, local governments and the Health and Safety Executives (HSE) all have a role in ensuring safety compliance [21]. The Council posited that the involvement of local governments strengthens health and safety inspection, helps prevent accidents by providing advice, investigates claims, and strengthens enforcement. [22] indicated that LGs must be involved in health and safety.

Health and safety in the Ghanaian construction industry is enforced through the use of fragmented laws, namely the Factories, Offices and Shops Act 1970 (ACT 328) and the Labour Act 2003 (Act 651) [15]. These general laws apply to all sectors and not only the construction industry. Sections 6 to 8, 10 to 12, 19, 20, 21, 25 to 30, 34 to 40, 43 to 54, 57, and 60 to 87 of the Factories, Offices and Shops Act apply to all building operations and works of engineering construction [23]. The Labour Act 2003 (Act 651) captures occupational health and safety issues under Part XV. This part of the Act deals with general health and safety conditions, exposure to imminent hazards, employer reporting occupational accidents and diseases, and specific measures [24]. The 1992 Constitution of the Republic of Ghana, under Chapter 5, Fundamental Human Rights and Freedom, Article 24(1), which deals with economic rights, stated, “Every person has the right to work under satisfactory, safe, and healthy conditions and shall receive equal pay for equal work without distinction of any kind.” The Constitution again states in Chapter 6, The Directive Principles of State Policy, Article 36(10), on Economic Objectives, that “The State shall safeguard the health, safety, and welfare of all persons in employment and shall establish the basis for the full deployment of the creative potential of all Ghanaians” [25]. Though there are very salient details in these two acts and the 1992 Constitution, there is gross laxity in their enforcement and implementation by the state and mandated state agencies [[13], [14], [15]]. The DFI is a department required by Act 328 of 1970 to regularly conduct technical safety, health, and welfare inspections of all premises within its jurisdiction to ensure occupational safety and health compliance. DFI is supposed to provide national leadership in occupational health and safety to prevent occupational accidents and diseases, thereby promoting and ensuring compliance with Act 328. However, the department has only ten regional offices out of the 16 regions. It is visible in the following regions: Ashanti - Kumasi, Western - Takoradi, Eastern - Koforidua, Northern - Tamale, Brong Ahafo - Sunyani, Upper East - Bolgatanga, Volta - Ho, Central - Cape Coast, Greater Accra - Tema, and Greater Accra - Accra [26]. [27] revealed in their study that DFI was not in every district. Unfortunately, the situation has not changed after 13 years. At the regional level, where they claim visibility, they lack commitment and only undertake periodic and selective inspections. The department lacks human and material resources [14]. DFI does not have a system to assess contractors' risk management strategies before project commencement, offers no auditing of their occupational health and safety (OHS) activities, and does not provide any form of training for firms, employees, master craftsmen, or trainees. The work of DFI would be more efficient if their presence could be felt in all districts [27], but that would take years to materialise and expose the lives of well-meaning construction employees to more danger. Based on these constraints, it will be more appropriate for DFI to cede some of their responsibilities to the local governments or establish offices at the district level and furnish them to oversee occupational health and safety (OHS) issues at the local government level. Alternatively, local governments, as institutions with the power to make bylaws [19], can capitalise on that to initiate some beneficial laws to support the effort of DFI to deal with OHS issues and sanitise the construction sector's health and safety-related topics.

In the UK, the law considers local government institutions integral parts of the regulatory system, contrary to Ghana's situation. Section 18 of the Health and Safety at Work Act 1974 gives every local area the power to make adequate provision for health and safety. The law ensures that LGs are directly involved in the governance arrangements for health and safety. Notwithstanding, local government institutions in Ghana can also leverage the provisions in the Local Government Act 2016 (Act 936). Against this backdrop, the study seeks to identify the local government variables that could help influence and shape health and safety performance in the Ghanaian construction industry and develop a confirmatory factor model using these variables.

## 2 Local government

**Fig. 1:**
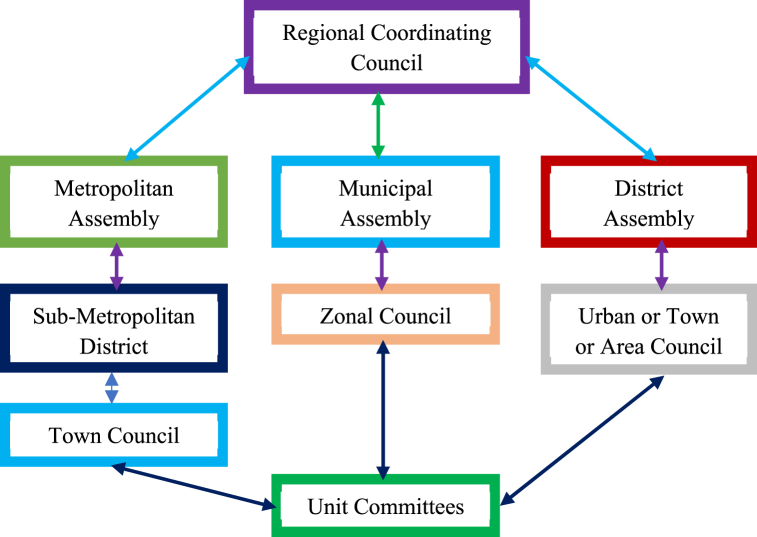
Local government structure in Ghana (Source: Forkuor & Adjei, 2016; Friedrich-Ebert-Stiftung Ghana, 2010).

Local government (LG) institutions in Ghana are public administrative institutions created by the 1992 constitution [28,29] with powers over local areas within the state [[29], [30], [31]] that play a vital role in the political and administrative authority of the local areas under their jurisdiction [19,32]. Local government institutions (LGs) under Section 12(1) are also responsible for the economic promotion of their local areas, the provision of guidance and direction, the supervision of other administrative authorities, planned, legislative, and executive functions, the overall development of the local area, and public health and safety, among others. The complete details of the functions of LGs are provided in Sections 12 and 13 of the Local Government Act of 2016. The Ghanaian spirit of local governance predates colonialism [32]. The 1992 Constitution, in Chapter 20: Decentralisation and Local Government, Article 240(1) on Local Government, states that “Ghana shall have a system of local government and administration which shall, as far as practicable, be decentralised” [19]. Ghana's Local government institutions are responsible for public health, environmental protection, sanitation, and education [33]. The local governance system was established to facilitate growth through collaboration between the central government and its numerous local areas [34]. Currently, Ghana is made up of 16 administrative regions with 261 districts [35,36]. These include six metropolitan assemblies, 96 municipal assemblies, and 159 district assemblies headed by metropolitan, municipal, and district chief executives [36]. The regional coordinating council sits at the top of the local governance structure, as illustrated in Fig. 1. The composition of assemblies according to section 5 of the Act comprises the metropolitan, municipal, or district chief executives, one person from each electoral area, member or members of parliament from the constituencies that fall within the area of authority of the district assembly, other members (shall not exceed thirty percent of the total membership of the district assembly appointed by the president in consultation with the traditional authorities and other interest groups in the district), a member of Parliament (counted among the seventy percent in the calculation of the thirty percent of the appointed members who shall have no voting rights), and District Co-ordinating Director as the secretary to the District Assembly appointed under section 75 (District Co-ordinating Director) of the Local Government Act [19]. Currently, local government institutions have no prescribed role concerning health and safety in construction. Section 104 of Act 936 makes provision for LGs through the District Planning Authority to enact building bylaws to control the construction of buildings, streets, hoardings, fences, and signboards; execute work in relation to existing buildings, structures, and streets; make provision for drainage and sanitation; the removal of abatement of obstructions and nuisance; and any other matter that may need guidance [19,32]. This section does not address construction health and safety (H&S). Section 14, which talks about health, is only related to public health and is not specific to H&S in construction.

According to Refs. [[37], [38], [39], [40]], local governments or authorities have direct responsibility for monitoring and enforcing the law. In the UK, the US, and within the EU, local governments have some form of responsibility, but in Ghana, the law gives them no leverage to help enforce health and safety in construction. The only avenue for LGs involvement would be through the introduction of bylaws. [41] opined that accidents, fatalities, and deaths in the US have reduced but not eradicated, even with enforcement in every federal state. This means that with local government efforts in Ghana, health and safety in the construction industry could be improved.

As an industry with many negligent employers and employees [42], there is a need to explore local government efforts that could enhance the construction industry's health and safety and protect the lives of the many Ghanaians who make a living in the industry within their districts and beyond. [43] indicated that one way local governments can contribute to enhancing health and safety and improving firms' organisational safety culture is to introduce sanctions for breaches. Health and safety laws, apart from being regulated by mandated state institutions, should also become a duty for LGs to control firms that operate within their districts, with the sole aim of improving safety.

The campaign for a hazardous-free construction work environment cannot be placed in the hands of only contractors; there is a need to complement their efforts and those of other stakeholders with the law and enforcement. In the area of enforcement and monitoring, the study seeks to explore how the local government institutions in Ghana can be fully integrated into construction work environment policing to enhance H&S performance. The seven LG roles identified and conceptualised to influence safety performance are explained below.

1. Assess contractor's and supplier's safety policies and risk management strategies – This intervention is to ensure that contractors and suppliers who are awarded contracts within a local jurisdiction have their safety policies and risk management strategies thoroughly assessed and verified to see if those strategies are complete to deal with the specific project risks. [43] stated that risk management strategies are structured ways of handling the dangers posed by the numerous hazards on site.
2. Assess firm's OHS structures prior to the commencement of site works – The assurance of good safety practices could be determined by the safety structures put in place by firms or private contracting individuals (PCIs). According to Ref. [44], the assessment of OHS structures will change the attitude of contractors toward health and safety.
3. Institute local government OHS approval and certification for new projects – Certification affirms the safety worthiness of firms or PCIs [45] after assessing their in-house policies and structures to deal with the challenges of a particular project. Project-based certification rather than something reviewed annually is preferred.
4. Monitor and audit firm's OHS activities during project delivery – Monitoring and auditing interventions ensure that the activities of firms and PCIs are monitored and audited regularly until the project is completed. Experts on monitoring duties can unearth the state of safety when they interact with workers. Reporting of all incidents must be compulsory so that during monitoring visits, an audit can be undertaken to see if there are any safety infringements. The state of health and safety can be attributed to a lack of monitoring by public agencies [46,47]. According to Ref. [48], monitoring is a vehicle for safety improvement.
5. Create OHS departments and committees that are adequately resourced to assist the Department of Factories Inspectorate – The effectiveness of monitoring and auditing is based on having the proper backing from bylaws and the needed human and material resources.
6. Develop sanctions for violating OHS statutory obligations – Though sanctions are not recommended as the first resort to an action of breach, they help shape and instil discipline in a system. Sanctions serve as a deterrent to health and safety breaches and can come in the form of fines, blacklisting, or cautions plus fines.
7. Register and organise OHS programmes for master craftsmen and trainees to sensitise them – Safety awareness and training are beneficial, as noted by Refs. [[49], [50], [51]]. They enlighten and influence individuals' perceptions.

## 3 The Ghanaian construction industry

The Ghanaian construction industry (GCI) is one of the essential industries in the country, and its role cannot be underestimated because it contributes immensely to the growth and the gross domestic product (GDP) [[52], [53], [54], [55]]. [56] referred to it as the engine of growth. It is a complex industry with many direct and indirect stakeholders and regulators [57]. It comprises building and civil engineering contractors, plumbing and engineering contractors, and other specialised contractors [58]. [59] postulated that the industry engages about 320,000 people and 1500 active building and construction contractors. Between 2006 and 2007, the Institute of Statistical Social and Economic Research (ISSER) posited that the construction industry contributed 0.7% and 1.0% to the nation's GDP [60]. Its contribution steadily increased over five years (2014–2019) from GHS12,183 to GHS21,013 [61]. The industry's performance makes it a core developmental component of the country's economy [5,62]. GCI comprises actors such as clients, contractors, consultants, stakeholders of different kinds, shareholders, the Ghana Chamber of Construction Industry (GHCCI), and regulators [52]. African Business Information reported that GCI was worth about US$5 billion. However, the most current market size of the industry, as valued in 2021, was US$13.2 billion, and it is projected to reach an average annual growth rate of more than 4% between 2023 and 2026 [63]. This makes the industry a critical component of Ghana's national development plan [59].

## 4 Health and safety performance

Health and safety in construction still enjoys global attention because of the delicate nature of the subject [4]. This is a result of the poor record of the construction industry and the notoriety it has gained over the years as the riskiest working environment. The death of 2.3 million workers yearly due to occupational accidents [7] indicates that H&S is being taken for granted. According to Ref. [64], the need for improvement in health and safety is long overdue. [65] viewed health and safety performance as a firm's performance in relation to its health and safety objectives or as an organisation's successes in safety [66]. According to Ref. [67], health and safety performance is “a measure of the level of effectiveness of those business activities aimed at the prevention of injury and disease to persons in the workplace.” [68] posited that the health and safety concept seeks to maintain workplace safety through varied planned interventions. [69] avowed that good safety performance does not depend on reactive but proactive approaches. They indicated that reactive methods become necessary when proactive strategies fail to produce the needed results. Safety improvement is not an overnight event; it thrives on good safety practices [70]. Many factors can influence health and safety; one such factor is the non-appreciation of stakeholders' responsibilities and roles, as revealed by Ref. [5]. [[49], [71]] highlighted a lack of health and safety intervention and awareness, non-adherence to H&S roles by project managers, a lack of leadership from clients and their representatives, neglect of H&S from inception through the contractor selection stage, organisational safety leadership problems, a lack of management commitment, and negligence by contractors. More than a decade ago [14,27], discovered that laxity on the part of state agencies mandated to enforce H&S laws and the underresourced nature of such agencies are some of the factors influencing H&S performance in the Ghanaian construction industry. The same was revealed by Ref. [12] after many years. They further identified the lack of adequate H&S policies, education, and sanctions as factors affecting H&S in Ghana's construction sector. However, [72] posited that to enhance performance in H&S, the efforts of all relevant stakeholders would have to be harnessed. H&S performance, according to Ref. [15], must be the collective responsibility of those involved in the whole process of project implementation. [50] averred that the quest for improved safety standards requires awareness creation. [73] reported that there is a direct, positive, and moderate relationship between health and safety management and H&S performance. Management is the set of practices that lead to performance. A positive set of practices correlates with positive performance. [12], however, identified safety education as a tool for advancing health and safety performance. [51,74] concluded that maintaining health and safety performance depends on management commitment, employee safety training, safety knowledge sharing, and several mediating and moderating variables. The study by Ref. [51] revealed an indirect relationship between management commitment and safety performance. This conclusion buttresses the findings of [75]. Bayram concluded that employee satisfaction also has a significant direct positive impact on health and safety performance, which confirms the revelations made by Refs. [76,77]. [2] posited that improved health and safety in the construction industry is indispensable for economic gains and productivity enhancement. [51] postulated that solid top management commitment is necessary for safety performance, even under vigorous safety enforcement by relevant agencies [78]. [78] proposed that poor safety performance is the product of a null management commitment to safety.

## 5 Methodology

The positivism philosophy was adopted for the study, and the approach considered suitable for this philosophy was deductive because it is highly structured and depends heavily on using large samples and measurements to analyse a range of data [79]. It is also more logically driven and objective [80]. The positivist philosophy is typically quantitative [81]. The researchers used specific quantitative variables developed with the help of literature as local government factors [82]. This was meant to measure and test causal hypotheses [[83], [84], [85]].

The quest for highly statistical data led to adoption of a quantitative method [79]. A survey was the most appropriate strategy for the selected method. Variables identified were developed into a questionnaire to generate data for determining their relative importance and unidimensionality, reliability, and the development of a confirmatory factor analysis (CFA) model [83,86]. This was done to aid in the explanation of the postulated concept of how local governments (LGs) could be partnered to improve health and safety performance in the Ghanaian construction industry (GCI) [84, 87).

The questionnaire was developed on a five-point scale ranging from 1 to 5, where 1 = very low influence, 2 = low influence, 3 = average influence, 4 = high influence, and 5 = very high influence. This is because reliability increases as scale points increase. Cross-sectional reliability increases from 2- to 3- to 5-point. The designed questionnaires were distributed to respondents to complete them to aid the data analysis process [87].

The study relied on the varied opportunities available in present-day survey studies [88]. Questionnaires in this study were administered using emails, WhatsApp platform, and Google Forms and by delivering hard copies to respondents who felt comfortable using them.

The study population was 7925, an all-inclusive population comprising contractors, architects, quantity surveyors, professional engineers in good standing with their professional associations, and lecturers from Technical Universities. The population of research gives it credence, reliability, and validity, hence, the involvement of these diverse professionals.

In order to generalise the findings of the study within the Ghanaian context, a sample was drawn from the total population using a probability sampling technique. The samples drawn were not proportional to individual sub-populations because the study's goal was not to compare the responses of the various professionals but to obtain a single view from professionals on the subject matter. The issue of sample size has always been a concern in research, but [89] posited that there is no absolute answer as to the size of a sample because sample sizes can be affected by a number of factors. However [88], prefer larger samples because they provide better impressions and determine the extent of generalisation. They advised researchers to find the most feasible way to determine appreciable sample sizes. [90] proposed a sample size of 400 for populations beyond 5000 and averred that sample size becomes immaterial when considering a population size over 5000. According to Ref. [91], robust and complex models should always have sample sizes above 200. Based on the guidelines for sample size selection proposed by Ref. [90], about populations above 5000, a sample size of 635 was deemed feasible to represent the all-in population. The study selected 235 more samples than the 400 proposed by the authors to address some of the infractions and non-responses that come with survey research. In addition, the selected sample size was to ensure that the minimum required sample needed to perform a robust analysis was achieved. A simple random sampling approach was used to select the subjects of the chosen sample size. The goal was to give all the subjects an equal opportunity to be selected. All the samples were selected one after the other without replacement, and at the end of the sampling, (202) engineers, (152) quantity surveyors, (142) contractors, (76) lecturers, and (63) architects were selected and used for the study. These categories of professionals were considered appropriate for this study because they operate in the construction industry and are familiar with the H&S challenges. Therefore, the researchers believed they could provide insight into what local government institutions could do to enhance safety in the Ghanaian industry based on their experiences as practitioners. The increase in the sample size was done to increase the response rate, improve the results, and allow for broader generalisation of the study results.

Descriptive statistics were employed in the analysis of respondents’ bio-data. The factors were first analysed using the relative importance index (RII) formula below, as given by Ref. [92].

$$RII=\frac{1n_{1}+2n_{2}+3n_{3}+4n_{4}+5n_{5}}{5\left(n_{1}1+n_{2}+n_{3}+n_{4}+n_{5}\right)}$$

where:

n_1_ = the number of respondents who chose very low influence

n_2_ = the number of respondents who chose low influence

n_3_ = the number of respondents who chose average influence

n_4_ = the number of respondents who chose high influence

n_5_ = the number of respondents who chose very high influence.

Using multiple predictors, the relative importance index (RII) was used to determine the relativity of local government-related factors that could influence health and safety in the construction industry [[93], [94], [95]]. Factors that recorded higher RIIs indicated higher cause or impact [96]. The index ranged from 0 to 1, with indices closer to 1 deemed high significance [96,97]. The RII values were classified into five different levels of importance, namely, high (0.8 ≤ RII ≤1), medium-high (0.6 ≤ RII ≤0.8), medium (0.4 ≤ RII ≤0.6), medium-low (0.2 ≤ RII ≤0.4) and low (0 ≤ RII ≤0.2) [98,99].

Multivariate analysis was performed using exploratory factor analysis (EFA) and confirmatory factor analysis (CFA). The assessment of the unidimensionality and reliability of the local government factors was performed using exploratory factor analysis (EFA) [100], while confirmatory factor analysis (CFA) was employed to validate the relationship between the manifest and latent variables. As the first step in structural equation modelling (SEM), the CFA was used for fit analysis [101]. The RII was used to determine the relative significance of the factors. However, the multivariate approach was used to determine the dimensionality and reliability of the factors and the construct they sought to measure and validate any existing inherent relationships. Multivariate analysis was used to determine the impact of local government factors on the variability of health and safety performance [102]. The multivariate correlational analysis EFA was performed using SPSS version 26, while AMOS version 22 was used for the confirmatory factor analysis (CFA). The robustness of the proposed CFA model was assessed using the information in Table 1.

**Table 1:** Indices for robust assessment.

Fit Index	Cut-off value	Comment	Source
S – Bχ^2^			[101,103,104,105,106,118119]
*Df*	0≥	Acceptable	
CFI	0.90≥ acceptable 0.95≥ good fit	Good fit	
PCFI	Less than 0.80	Good fit	
RMSEA	Less than 0.08	Acceptable	
RMSEA 95% CI	0.00–0.08 “good fit”	Acceptable	
NFI	Greater than 0.95 “good fit”	Good fit	
IFI	Greater than 0.90 “good fit”	Good fit	
PNFI	Less than 0.80	Good fit	
RMR	Less than 0.05 “good fit”	Good fit	
GFI	Greater than 0.90 “good fit”	Good fit	

## 6 Data analysis and results

### 6.1 Background information of respondents

The total number of respondents whose responses were analysed included 134 quantity surveyors, 125 engineers, 84 contractors, 62 lecturers, and 49 architects. In all, 512 responses were received, but only 454 were valid. Hence valid responses were used for the analysis. Data collected revealed that 126 had 6–10 years, 103 had 11–15 years, 101 had 16–20 years, 68 out of the total had 2–5 years of working experience, and 56 possessed 21 years and above experience. The highest qualification among the respondents who participated in the study was a Ph. D. Out of the total number, only 32 had it. The second highest qualification was a master's degree which 154 respondents possessed, 176 had a first degree, and 85 had Diploma/Higher National Diploma (HND). However, 7 possessed technician certificates.

### 6.2 Relative importance index (RII) - local government factors

The relative importance index and their accompanied rankings of the seven (7) local government-related factors that could affect health and safety performance in the Ghanaian construction industry (GCI) are presented in Table 2 below. The variable “develop sanctions for violating OHS statutory obligations” with factor code (LGRF6) was ranked the most important factor among the seven factors. This recorded an RII of 0.912. The second most important factor was “institute local government OHS approval and certification for new projects (LGRF3),” with an RII of 0.909. The third and fourth were “create OHS departments and committees that are adequately resourced to assist the Department of Factories Inspectorate (LGRF5)” and “monitor and audit firms' OHS activities during project delivery (LGRF4),” with RIIs of 0.906 and 0.897, respectively. The fifth rated factor was “assess contractor's and supplier's safety policies and risk management strategies (LGRF1)”. This variable registered an RII of 0.896. “Register and organise OHS programmes for master craftsmen and trainees to sensitise them (LGRF7)” was considered the sixth (6th) with an RII of 0.894, while “assess firm's OHS structures prior to the commencement of site works (LGRF2)” was ranked seven (7th) with an RII of 0.885. The relative importance indices for all seven local government-related factors were classified as high since they were above 0.8 [98,99]. The ranking shows that respondents found sanctioning culpable firms for H&S breaches as the most important factor that could contribute to performance improvement in health and safety in the construction industry. However, they considered the assessment of firm's OHS structures before the commencement of site works as something that had minor relative importance.

**Table 2:** RII of local government factors that could affect health and safety performance.

Factor Code	Local Government Related Factors	RII	Rank
LGRF6	Develop sanctions for violating OHS statutory obligations.	0.912	1st
LGRF3	Institute local government OHS approval and certification for new projects.	0.909	2nd
LGRF5	Create OHS departments and committees that are adequately resourced to assist the Department of Factories Inspectorate.	0.906	3rd
LGRF4	Monitor and audit firm's OHS activities during project delivery.	0.897	4th
LGRF1	Assess contractor's and supplier's safety policies and risk management strategies.	0.896	5th
LGRF7	Register and organise OHS programmes for master craftsmen and trainees to sensitise them.	0.894	6th
LGRF2	Assess firm's OHS structures prior to the commencement of site works.	0.885	7th

### 6.3 Exploratory factor analysis: Dimensionality of local government-related factors (LGRF) construct

The exploratory factor analysis examined the factor structure and internal consistency of the manifest variables associated with the local government-related factors (LGRF) construct. The analysis used Maximum Likelihood with Varimax rotation (ML Varimax). The results revealed that all seven items measuring the construct demonstrated consistency. The Kaiser-Meyer-Olkin (KMO) measure obtained was 0.916, surpassing the recommended threshold of 0.70, and Bartlett's test of sphericity yielded a significance level of p < 0.000, indicating that factor analysis was appropriate for the data. The factor loadings were assessed using a threshold of 0.5, based on [107], which was considered better than the recommended value of 0.40 suggested by Ref. [108]. All seven items exceeded the 0.50 threshold and were loaded as a single component, labelled “local government effort” (LGE), as shown in Table 3. After the multivariate EFA analysis, the corrected item-total correlation for the component (LGE) was examined using the suggested cut-off value 0.30. The Cronbach's alpha obtained for the component was 0.910, surpassing the value of 0.800. This indicates that the component (LGE) variables exhibited good internal reliability [109]. In all, the results from the factor analysis support the one-dimensionality and reliability of the local government-related factors, as presented in Table 4. The findings provide valuable insights into the consistency and robustness of the measures used in assessing the LGRF construct.

**Table 3:** Local government health and safety performance factors.

Local government factors	Component
	1
Assess contractor's and supplier's safety policies and risk management strategies.	0.820
Assess firm's OHS structures prior to the commencement of site works.	0.795
Institute local government OHS approval and certification for new projects.	0.790
Monitor and audit firm's OHS activities during project delivery.	0.767
Create OHS departments and committees that are adequately resourced to assist the Department of Factories Inspectorate.	0.759
Develop sanctions for violating OHS statutory obligations.	0.729
Register and organise OHS programmes for master craftsmen and trainees to sensitise them.	0.716

**Table 4:** Unidimensionality and reliability of local government effort construct.

Local government factors	Local government effort (LGE)	Corrected Item-Total Correlation	Squared Multiple Correlation	Cronbach's Alpha if Item Deleted	Cronbach's Alpha
Assess contractor's and supplier's safety policies and risk management strategies.	0.820	0.775	0.612	0.891	0.910
Assess firm's OHS structures prior to the commencement of site works.	0.795	0.747	0.595	0.894	
Institute local government OHS approval and certification for new projects.	0.790	0.745	0.580	0.894	
Monitor and audit firm's OHS activities during project delivery.	0.767	0.732	0.553	0.896	
Create OHS departments and committees that are adequately resourced to assist the Department of Factories Inspectorate.	0.759	0.721	0.547	0.897	
Develop sanctions for violating OHS statutory obligations.	0.729	0.694	0.511	0.900	
Register and organise OHS programmes for master craftsmen and trainees to sensitise them.	0.716	0.678	0.476	0.902	

### 6.4 Structural equation model (SEM) for local government-related factors (LGRF) construct

After confirming the one-dimensionality and reliability of the construct through Exploratory Factor Analysis (EFA), Confirmatory Factor Analysis (CFA) was conducted. The CFA employed a three-statistical strategy of fit indices, as recommended by Ref. [104], before assessing the goodness of fit for the local government-related factors (LGRF) construct. The sample data for the LGRF model yielded an S-Bχ2 value of 4.665 with 14 degrees of freedom (df) and a probability (p) of 0.0000. According to Ref. [110], chi-square values of 5 or less can be considered benchmarks. The chi-square value indicated that the sample data significantly deviated from the postulated model, implying a good fit. The Comparative Fit Index (CFI) value was 0.973, exceeding the cut-off threshold 0.95 recommended by Refs. [104,111]. Therefore, the model could be described as well-fitting or acceptable [111]. It is worth noting that CFI, as an accurate estimator, should not exceed a theoretical maximum of 1 [112]. The Normed Fit Index (NFI) value was 0.964, surpassing the recommended cut-off of NFI ≥0.95, which aligns with the model being considered well-fitting [[105], [111], [112]]. However, the Parsimony Normed Fit Index (PNFI) value was 0.643, falling below the cut-off value of 0.80, indicating a good fit [105]. The Root Mean Square Residual (RMR) value obtained was 0.014, less than 0.05, suggesting a good fit [101,112,105]. Similarly, the goodness-of-fit index (GFI) recorded a value of 0.962, greater than 0.090 as recommended by Refs. [104,[105], [106], [111], [112]], further supporting a favourable fit. These fit indices for the LGRF model suggested that the hypothesised model adequately describes the sample data and could be included in the full latent variable model analysis. Refer to Table 5 for a presentation of the fit indices for the local government-related factors.

**Table 5:** Robust fit indices for local government effort (LGE).

Fit Index	Cut-Off Value	Estimate	Comment
S – Bχ^2^		4.665	
*Df*	0≥	14	Acceptable
CFI	0.90≥ acceptable 0.95≥ good fit	0.973	Good fit
PCFI	Less than 0.80	0.648	Good fit
RMSEA	Less than 0.08	0.072	Acceptable
RMSEA 95% CI	0.00–0.08 “good fit”	0.069–0.081	Acceptable
NFI	Greater than 0.95 “good fit”	0.964	Good fit
IFI	Greater than 0.90 “good fit”	0.972	Good fit
PNFI	Less than 0.80	0.643	Good fit
RMR	Less than 0.05 “good fit”	0.014	Good fit
GFI	Greater than 0.90 “good fit”	0.962	Good fit

**Fig. 2:**
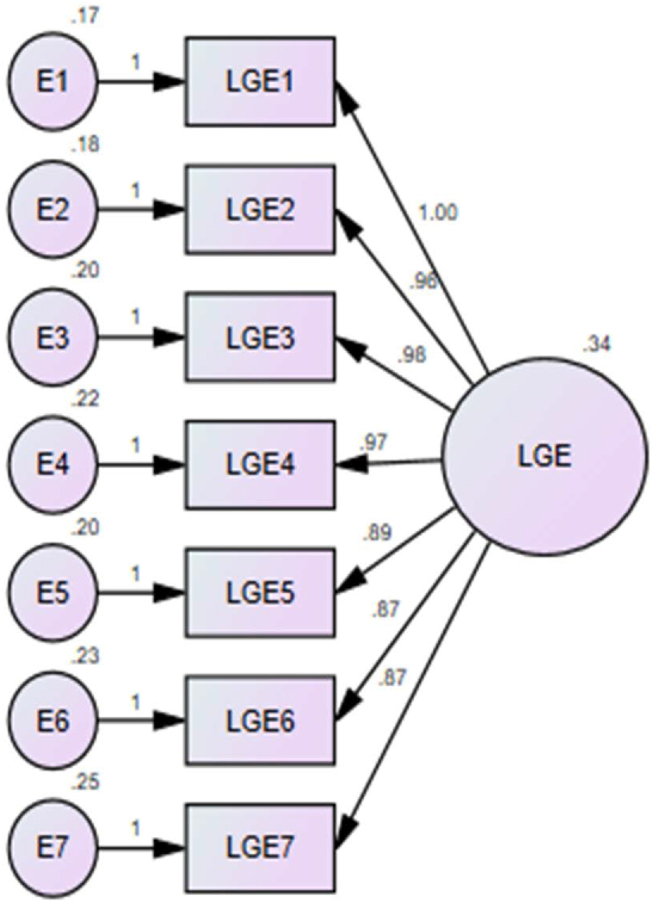
CFA model for local government effort.

Fig. 2 and Table 6 present the features of the unidimensional model for the Local Government-Related Factors (LGRF). The final Confirmatory Factor Analysis (CFA) analysis included all seven indicator variables [113], which were identified as LGE (LGE1, LGE2, LGE3, LGE4, LGE5, LGE6, and LGE7) from the 454 cases analysed for this construct.

**Table 6:** Final conceptual model indicator variables for local government effort (LGE).

Latent Component	Indicator Variable	Measurement Variable	Label
LGE		Assess contractor's and supplier's safety policies and risk management strategies.	LGE1
		Assess firm's OHS structures prior to the commencement of site works.	LGE2
		Institute local government OHS approval and certification for new projects.	LGE3
		Monitor and audit firm's OHS activities during project delivery.	LGE4
		Create OHS departments and committees that are adequately resourced to assist the Department of Factories Inspectorate.	LGE5
		Develop sanctions for violating OHS statutory obligations.	LGE6
		Register and organise OHS programmes for master craftsmen and trainees to sensitise them.	LGE7

Table 7 reveals that all correlation values were below 1.00, and all p-values were lower than the significance level of 0.05. Hence, the estimates can be considered both reasonable and statistically significant. Notably, variable LGE1 displayed the highest standardised coefficient of 0.820, implying that the “assessment of contractor's and supplier's safety policies and risk management strategies by local governments” might considerably impact health and safety performance in the construction industry.

**Table 7:** Factor loading and p-value of local government effort (LGE).

Hypothesised relationships (Path)	Unstandardised Coefficient (λ)	Standardised Coefficient (λ)	P-Value	R Squared	Significant at 5% Level
LGE1 ← LGE	1.000	0.820	0.00	0.673	Yes
LGE2 ← LGE	0.961	0.795	0.00	0.632	Yes
LGE3 ← LGE	0.984	0.790	0.00	0.624	Yes
LGE4 ← LGE	0.969	0.767	0.00	0.589	Yes
LGE5 ← LGE	0.890	0.759	0.00	0.576	Yes
LGE6 ← LGE	0.8741	0.729	0.00	0.532	Yes
LGE7 ← LGE	0.870	0.716	0.00	0.513	Yes

The majority of the parameter estimates displayed high correlation values, approaching 1.00. These high correlation values indicate a strong linear association between the indicator variables and the unobserved variables (LGE). Additionally, the R Squared values were also in close proximity to the desired value of 1.00, indicating that the factors account for a substantial amount of the variance in the indicator variables. Consequently, the results strongly imply that the indicator variables effectively predict the unobserved components since all the measured variables are significantly associated with the component (LGE) within the context of local government-related factors.

## 7 Discussion of results

The study employed three different approaches to analysing the data. The first was to determine the order of importance of the factors, which was undertaken using RII; the second was to identify the unidimensionality and reliability of the LG factors; and the third was to determine model fitness by verifying the factor structure. The RII obtained by each variable revealed that the three most important LG variables that could improve H&S in the Ghanaian construction industry are “develop sanctions for violating OHS statutory obligations,” “institute local government OHS approval and certification for new projects,” and “create OHS departments and committees that are adequately resourced to assist the Department of Factories Inspectorate.” The least was “assess firm's OHS structures prior to the commencement of site works.” The variable was considered the least important by ranking but was still regarded as high. According to Refs. [98,99] categorisation (0.8 ≤ RII ≤1), all the LG variables are highly important. [114] posited that sanctions are necessary to ensure compliance, but certification confirms good practices [45].

The EFA multivariate analysis, on the other hand, considered “assess contractor's and supplier's safety policies and risk management strategies” as the most significant LG variable. This was followed by “assess firm's OHS structures prior to the commencement of site works,” “institute local government OHS approval and certification for new projects,” “monitor and audit firm's OHS activities during project delivery” “create OHS departments and committees that are adequately resourced to assist the Department of Factories Inspectorate” in that order as indicated in Table 2. The LG variables’ Cronbach alpha (0.910) revealed good internal reliability and unidimensionality.

The development of the CFA model was to provide valuable information on the fitness of the dataset [101]. The postulated model (Fig. 2) could be described as a good fit because it sufficiently describes the sample data. The study revealed that the model developed using LG variables was a one-factor model. The correlated values and p-values were indicative of reasonable statistical significance. CFA, as an advanced statistical technique, revealed that variable LGE1 (“assess contractor's and supplier's safety policies and risk management strategies”) with a standardised coefficient of 0.820 is the LG factor that can significantly improve health and safety performance. Safety policy was validated as a useful safety programme element for championing improved health and safety [1,[115], [116], [117]]. Regarding the assessment of risk management strategies, [118,119] indicated that risk is a critical success factor for moderating risks and enhancing safety performance improvement. [120,103] posited that assessing risk management strategies influences safety improvement, risk safety, and risk control. This was followed by “assess firm's OHS structures prior to the commencement of site works” and “institute local government OHS approval and certification for new projects.” The performance of each factor based on its standardised coefficient can be found in Table 7.

## 8 Conclusions

The study sought to establish how local government roles could help influence health and safety performance in the Ghanaian construction industry (GhCI). This led to the development of seven (7) factors that the study found relevant. The study used the relative importance index to observe the most important factors that could improve health and safety performance in the industry. A multivariate statistical analysis was also performed to establish the structure of the factors and similarly verify them to determine how well the elements aligned with the hidden variable.

The study concluded, based on the relative importance indices obtained by the various factors, that “developing sanctions for violating OHS statutory obligations” was the most important factor that could enhance H&S. The institution of local government OHS approval and certification for new projects and the creation of OHS departments and committees that are adequately resourced to assist the Department of Factories Inspectorate were also found to be important factors that could shape the health and safety landscape in the industry. All seven factors were high according to the RII categorisation given by Refs. [98,99]. They can influence H&S in the Ghanaian construction industry (GhIC).

The calculated Cronbach's alpha indicated that the sample data was sufficiently reliable and unidimensional. The p-values and associated values also prove the predicted values were reasonable and statistically significant. The study showed that the CFA was a one-factor model for local government characteristics. According to the standardised coefficient of LGE1, evaluating suppliers' and contractors' safety policies and risk management plans could significantly impact health and safety performance.

While the relative importance index suggests that sanctions might be the best strategy to improve H&S in the industry, the multivariate analysis' projected numbers show that evaluating contractors' and suppliers’ safety policies and risk management plans is crucial. As a local government-related factor, this factor has a more significant impact. The multivariate analysis provides a better picture of the variables with critical effects on health and safety than the RII. According to Ref. [121], multivariate estimates are more realistic, improve reliability and validity, and aid in drawing more accurate conclusions.

The confirmatory factor analysis and the relative importance index revealed that local government effort (LGE) is key if H&S enhancement is to be realised in Ghana's construction industry. The study concludes that local government effort is required to improve H&S in the construction industry (CI). Theoretically, the study contributes to the body of knowledge on health and safety performance by identifying local government efforts that could instil some sanity into the H&S arena and the need to integrate DAs into future safety reforms. Furthermore, the study provides an excellent theoretical foundation for future studies investigating local government efforts concerning health and safety performance.

The practical implication of the study is that it identifies what LGs or DAs can do to help enhance health and safety in the industry. According to the study, LGs are essential stakeholders who can help sanitise the poor health and safety records of the construction industry. They can contribute their quota to health and safety improvement in the construction industry by instituting bylaws as empowered by the Local Government Act 2016 (Act 936) that are in tandem with the Factories, Offices and Shops Act 1970 (Act 328) and the Labour Act 2003 (Act 651) to reinforce the provisions of articles 24(1) and 36(10) in the 1992 Constitution of the state. Alternatively, the government, looking at the importance of H&S, can, as a matter of state policy, through legislation, cede some of the powers of the DFI to LGs or establish functional and resourced units within the DAs and give the DFI a regional oversight responsibility. LGs must employ people whose professions are relevant and related to the core business of the construction industry and must not engage in business as usual by employing political cronies whose specialisations may not be aligned with the subject area. When this happens, it will defeat the purpose of looking for solutions to the problem.

The factors used in this study were carefully modelled based on the nature of the Ghanaian construction industry landscape; therefore, care must be taken not to generalise its findings to other jurisdictions. In addition, the professionals selected were Ghanaian practitioners; thus, whatever is depicted in the study as findings reflect their opinions and experiences.

## Author contribution statement

Akomah Benjamin Boahene: Conceived and designed the experiments; Performed the experiments; Analysed and interpreted the data; Contributed reagents, materials, analysis tools or data; Wrote the paper. Prasanna Venkatesan Ramani, Ph.D.: Conceived and designed the experiments; Performed the experiments; Contributed reagents, materials, analysis tools or data; Wrote the paper.

## Data availability statement

No data was used for the research described in the article.

## Ethical declaration

All participants provided informed consent to participate in the study.

## Declaration of competing interest

The authors declare that they have no known competing financial interests or personal relationships that could have appeared to influence the work reported in this paper.
