# Supplementary material for: Local government institutions in Ghana: Core partners in health and safety performance in the construction industry
Source: Heliyon. 2023 Aug 24;9(9):e19423. doi: 10.1016/j.heliyon.2023.e19423 (PMC10472057; doi:10.1016/j.heliyon.2023.e19423)
Supplement: Multimedia component 1 [file mmc1.docx]

RESEARCH INTRODUCTION LETTER AND QUESTIONNAIRE

**
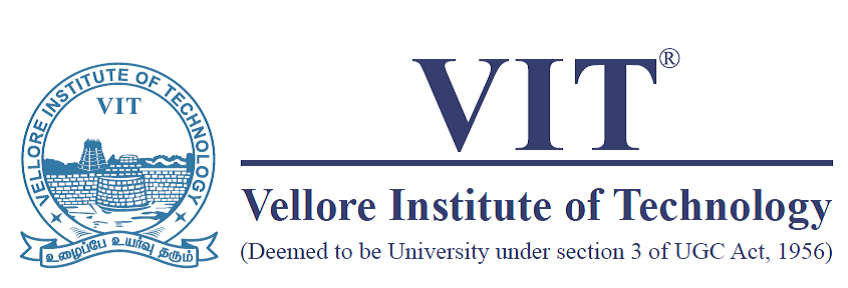
**

School of Civil Engineering, SCE

VIT University, India

Tamil Nadu 632014, India.

June, 2021

Dear Respondent

The Postgraduate School of Civil Engineering (SCE) at the VIT University is undertaking research aimed at developing a confirmatory factor model for local government health and safety factors that could influence OHS performance in the Ghanaian construction industry. We humbly request your contribution and effort to complete this questionnaire. It should take no longer than 5 minutes of your time. Your response is vital to the success of this study.

We would like to assure you that your response will be treated confidentially. As a first step towards protecting the confidentiality of your response, please do not enter your name or contact information on the questionnaire.

Please do not hesitate to contact us if you have any questions or concerns at akomah.benjamin2019@vitstudent.ac.in and prasanna.venkatesan@vit.ac.in

Thank you for your participation.

Akomah, Benjamin Boahene

Ph.D. Candidate

**QUESTIONNAIRE FOR**

**LOCAL GOVERNMENT-RELATED FACTORS THAT CAN INFLUENCE HEALTH AND SAFETY PERFORMANCE IN THE GHANAIAN CONSTRUCTION INDUSTRY**

**Please kindly answer the following questions by crossing (x) in the appropriate spaces provided.**

**SECTION A: PROFILE OF RESPONDENT**

1. Profession

| Engineer |  | Quantity surveyor |  | Architect |  | Contractor |  | Lecturer |  |
| --- | --- | --- | --- | --- | --- | --- | --- | --- | --- |

1. Respondent’s highest qualification

| Technician |  | Diploma/HND |  | Bachelors |  | Masters |  | Doctorate |  |
| --- | --- | --- | --- | --- | --- | --- | --- | --- | --- |

1. Respondent’s years of practice

| 2-5 years |  | 6-10 years |  | 11-15 years |  | 16-20 years |  | 21years and above |  |
| --- | --- | --- | --- | --- | --- | --- | --- | --- | --- |

**SECTION TWO: LOCAL GOVERNMENT OHS FACTORS**

1. **LOCAL GOVERNMENT RELATED FACTORS (LGRF)**

Below is a list of local government factors that could influence health and safety performance in the Ghanaian construction industry. Please kindly indicate the extent of the influence these factors would have on health and safety performance by crossing [**x**] in the appropriate box.

| **Code** | **Local Government Related Factors** | **Extent of Influence**  **[Low High]** | | | | |
| --- | --- | --- | --- | --- | --- | --- |
|  |  | **1** | **2** | **3** | **4** | **5** |
| LGRF 1 | Create OHS departments and committees that are adequately resourced to assist the Department of Factories Inspectorate. |  |  |  |  |  |
| LGRF 2 | Assess firms OHS structures prior to commencement of site works. |  |  |  |  |  |
| LGRF 3 | Institute local government OHS approval and certification for new projects. |  |  |  |  |  |
| LGRF 4 | Assess contractors’ and suppliers’ safety policies and risk management strategies. |  |  |  |  |  |
| LGRF 5 | Develop sanctions for violating OHS statutory obligations. |  |  |  |  |  |
| LGRF 6 | Monitor and audit firms OHS activities during project delivery. |  |  |  |  |  |
| LGRF 7 | Register and organise OHS programmes for master craftsmen and trainees to sensitise them. |  |  |  |  |  |
